# *N* ^2^,*N* ^2^,*N* ^5^,*N* ^5^-Tetra­kis(2-chloro­ethyl)-3,4-dimethyl­thio­phene-2,5-dicarboxamide

**DOI:** 10.1107/S1600536809052374

**Published:** 2009-12-12

**Authors:** Yi-Dan Tang, Rong-Xia Geng, Cheng-He Zhou

**Affiliations:** a School of Chemistry and Chemical Engineering, Southwest University, Chongqing 400715, People’s Republic of China

## Abstract

In the title compound, C_16_H_22_Cl_4_N_2_O_2_S, the two imide groups adopt a *trans* arrangement relative to the central thienyl ring, so the four terminal 2-chloro­ethyl arms adopt different orientations. In the crystal, mol­ecules are linked by weak C—H⋯Cl and C—H⋯O hydrogen bonds into a three-dimensional network.

## Related literature

For general background to nitro­gen mustard agents as anti­tumor dugs, see: Zhuang *et al.* (2008 ▶). For the synthesis, see: Luo *et al.* (2007 ▶). For a related structure, see: Dong *et al.* (2006 ▶).
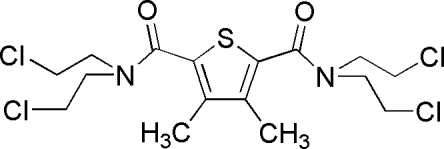


## Experimental

### 

#### Crystal data

C_16_H_22_Cl_4_N_2_O_2_S
*M* *_r_* = 448.22
Monoclinic, 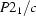

*a* = 7.9238 (4) Å
*b* = 21.1712 (11) Å
*c* = 12.6186 (7) Å
β = 99.2380 (10)°
*V* = 2089.39 (19) Å^3^
*Z* = 4
Mo *K*α radiation
μ = 0.68 mm^−1^
*T* = 298 K
0.25 × 0.22 × 0.20 mm

#### Data collection

Bruker APEXII area-detector diffractometer
Absorption correction: multi-scan (*SADABS*; Sheldrick, 1996 ▶) *T*_min_ = 0.849, *T*_max_ = 0.876
13412 measured reflections
4008 independent reflections
3342 reflections with *I* > 2σ(*I*)
*R*_int_ = 0.018

#### Refinement

*R*[*F* ^2^ > 2σ(*F* ^2^)] = 0.042
*wR*(*F* ^2^) = 0.126
*S* = 1.04
4008 reflections
228 parameters
H-atom parameters constrained
Δρ_max_ = 0.88 e Å^−3^
Δρ_min_ = −0.63 e Å^−3^

### 

Data collection: *APEX2* (Bruker, 2004 ▶); cell refinement: *SAINT* (Bruker, 2004 ▶); data reduction: *SAINT*; program(s) used to solve structure: *SHELXS97* (Sheldrick, 2008 ▶); program(s) used to refine structure: *SHELXL97* (Sheldrick, 2008 ▶); molecular graphics: *SHELXTL* (Sheldrick, 2008 ▶); software used to prepare material for publication: *APEX2* (Bruker, 2004 ▶) and *publCIF* (Westrip, 2009 ▶).

## Supplementary Material

Crystal structure: contains datablocks I, global. DOI: 10.1107/S1600536809052374/ng2703sup1.cif

Structure factors: contains datablocks I. DOI: 10.1107/S1600536809052374/ng2703Isup2.hkl

Additional supplementary materials:  crystallographic information; 3D view; checkCIF report

## Figures and Tables

**Table 1:** Hydrogen-bond geometry (Å, °)

*D*—H⋯*A*	*D*—H	H⋯*A*	*D*⋯*A*	*D*—H⋯*A*
C14—H14*B*⋯O2^i^	0.97	2.45	3.257 (3)	141
C14—H14*A*⋯Cl1^ii^	0.97	2.80	3.632 (3)	145
C6—H6*B*⋯O1^iii^	0.96	2.54	3.474 (3)	166
C5—H5*B*⋯O1^iv^	0.96	2.54	3.477 (3)	165

Symmetry codes: (i) 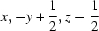; (ii) 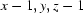; (iii) 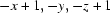; (iv) 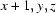.

**Fig. 1.**
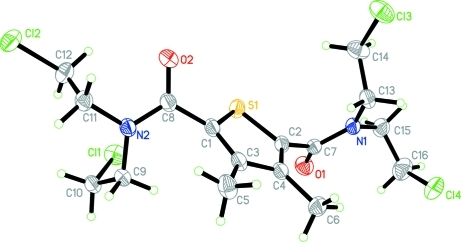
The molecular structure of the title compound, showing the atom-numbering scheme. Displacement ellipsoids are drawn at the 50% probability level.

**Fig. 2.**
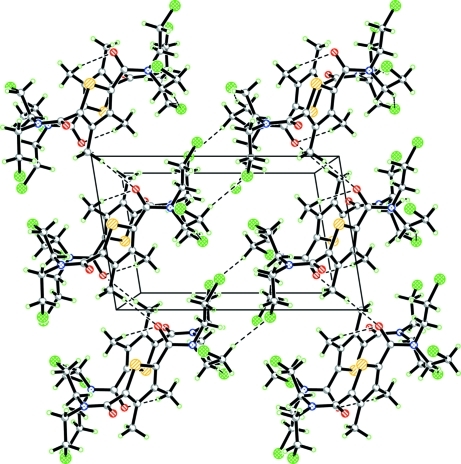
Packing diagram.

## supplementary crystallographic information

### Comment

Nitrogen mustard agents are one of the most important antitumor dugs, and have been widely used for the treatment of solid neoplastic and leukemia tumor for many years. The incorporation of amido and/or conjugated moiety into nitrogen mustards often helps to decrease the toxicity and improve the target affinity due to the dispersion of N atom electron atmosphere density (Zhuang *et al.*, 2008). Herein, in order to find new antitumor dugs, we have successfully synthesized the title compound (I) by an acylation reaction of bis(2-chloroethyl)amine wiht 3,4-dimethylthiophene-2,5-dicarbonyl dichloride (Luo *et al.*, 2007) and fully characterized by single-crystal X-ray diffraction.

The molecular structure of the title compound is shown in Fig. 1. Single crystal analysis revealed that two imide groups of the title compound adopt *trans*-conformation arrangement (Dong *et al., *2006) compared with the central thiophene ring, so the four terminal 2-chloroethyl arms are oriented in the different orientation. As indicated in Fig. 2, in the solid state, these moleculers are bonded together with Cl···H—C hydrogen bonds into an H-bonding-driven three-dimensional network, corresponding O(7)···H(3 A), O(7)···O(3), and O(7)···H(3 A)—O(3) data are 2.33 Å, 3.19 Å and 145.1°, respectively.

### Experimental

The title compound (I) was gained by amidation of 3,4-dimethylthiophene-2,5-dicarbonyl dichloride (1 mmol) with bis(2-chloroethyl)amine (2 mmol) according to literature (Luo *et al.*, 2007). A crystal of (I) suitable for X-ray analysis was grown from a mixture solution of ethyl acetate and petroleum ether by slow evaporation at room temperature.

### Refinement

Hydrogenatoms were placed in calculated positions with C—H =0.97Å (methylene) and 0.96Å (methyl) with *U*_iso_(H) = 1.2Ueq(*C*)(methylene C) or 1.5Ueq(C) (methyl C).

### Crystal data

**Table:**

C_16_H_22_Cl_4_N_2_O_2_S	*F*(000) = 928
*M**_r_* = 448.22	*D*_x_ = 1.425 Mg m^−^^3^
Monoclinic, *P*2_1_/*c*	Mo *K*α radiation, λ = 0.71073 Å
Hall symbol: -P 2ybc	Cell parameters from 7657 reflections
*a* = 7.9238 (4) Å	θ = 1.0–28.3°
*b* = 21.1712 (11) Å	µ = 0.68 mm^−^^1^
*c* = 12.6186 (7) Å	*T* = 298 K
β = 99.238 (1)°	Block, white
*V* = 2089.39 (19) Å^3^	0.25 × 0.22 × 0.20 mm
*Z* = 4	

### Data collection

**Table:**

Bruker APEXII area-detector diffractometer	4008 independent reflections
Radiation source: fine-focus sealed tube	3342 reflections with *I* > 2σ(*I*)
graphite	*R*_int_ = 0.018
φ and ω scan	θ_max_ = 26.0°, θ_min_ = 1.9°
Absorption correction: multi-scan (*SADABS*; Sheldrick, 1996)	*h* = −9→9
*T*_min_ = 0.849, *T*_max_ = 0.876	*k* = −26→26
13412 measured reflections	*l* = −15→15

### Refinement

**Table:**

Refinement on *F*^2^	Primary atom site location: structure-invariant direct methods
Least-squares matrix: full	Secondary atom site location: difference Fourier map
*R*[*F*^2^ > 2σ(*F*^2^)] = 0.042	Hydrogen site location: inferred from neighbouring sites
*wR*(*F*^2^) = 0.126	H-atom parameters constrained
*S* = 1.04	*w* = 1/[σ^2^(*F*_o_^2^) + (0.0694*P*)^2^ + 1.0128*P*] where *P* = (*F*_o_^2^ + 2*F*_c_^2^)/3
4008 reflections	(Δ/σ)_max_ < 0.001
228 parameters	Δρ_max_ = 0.88 e Å^−^^3^
0 restraints	Δρ_min_ = −0.63 e Å^−^^3^

### Special details

**Table:**

Geometry. All e.s.d.'s (except the e.s.d. in the dihedral angle between two l.s. planes) are estimated using the full covariance matrix. The cell e.s.d.'s are taken into account individually in the estimation of e.s.d.'s in distances, angles and torsion angles; correlations between e.s.d.'s in cell parameters are only used when they are defined by crystal symmetry. An approximate (isotropic) treatment of cell e.s.d.'s is used for estimating e.s.d.'s involving l.s. planes.
Refinement. Refinement of *F*^2^ against ALL reflections. The weighted *R*-factor *wR* and goodness of fit *S* are based on *F*^2^, conventional *R*-factors *R* are based on *F*, with *F* set to zero for negative *F*^2^. The threshold expression of *F*^2^ > σ(*F*^2^) is used only for calculating *R*-factors(gt) *etc*. and is not relevant to the choice of reflections for refinement. *R*-factors based on *F*^2^ are statistically about twice as large as those based on *F*, and *R*- factors based on ALL data will be even larger.

### Fractional atomic coordinates and isotropic or equivalent isotropic displacement parameters (Å^2^)

**Table:**

	*x*	*y*	*z*	*U*_iso_*/*U*_eq_	
C1	0.6440 (3)	0.16192 (11)	0.56639 (16)	0.0422 (5)	
C2	0.4663 (3)	0.10478 (11)	0.41970 (16)	0.0443 (5)	
C3	0.7314 (3)	0.11337 (11)	0.52879 (16)	0.0443 (5)	
C4	0.6267 (3)	0.07973 (11)	0.44327 (16)	0.0452 (5)	
C5	0.9129 (3)	0.09640 (14)	0.5718 (2)	0.0604 (7)	
H5A	0.9596	0.1267	0.6251	0.091*	
H5B	0.9788	0.0965	0.5142	0.091*	
H5C	0.9166	0.0551	0.6034	0.091*	
C6	0.6887 (4)	0.02243 (13)	0.3905 (2)	0.0622 (7)	
H6A	0.5947	0.0033	0.3441	0.093*	
H6B	0.7362	−0.0073	0.4445	0.093*	
H6C	0.7749	0.0348	0.3491	0.093*	
C7	0.3109 (3)	0.08236 (11)	0.34627 (17)	0.0458 (5)	
C8	0.7126 (3)	0.21071 (11)	0.64859 (17)	0.0422 (5)	
C9	0.6710 (3)	0.13373 (12)	0.79073 (18)	0.0500 (6)	
H9A	0.7502	0.1203	0.8534	0.060*	
H9B	0.6717	0.1022	0.7350	0.060*	
C10	0.4939 (4)	0.13895 (13)	0.8188 (2)	0.0643 (7)	
H10A	0.4128	0.1483	0.7547	0.077*	
H10B	0.4904	0.1732	0.8694	0.077*	
C11	0.7869 (4)	0.24259 (14)	0.8346 (2)	0.0575 (6)	
H11A	0.7587	0.2291	0.9031	0.069*	
H11B	0.7269	0.2819	0.8152	0.069*	
C12	0.9744 (4)	0.25440 (18)	0.8472 (3)	0.0832 (10)	
H12A	1.0070	0.2838	0.9059	0.100*	
H12B	1.0009	0.2738	0.7822	0.100*	
C13	0.4527 (3)	0.10073 (11)	0.18708 (17)	0.0459 (5)	
H13A	0.4924	0.0670	0.1451	0.055*	
H13B	0.5478	0.1132	0.2414	0.055*	
C14	0.4016 (3)	0.15598 (12)	0.11482 (18)	0.0504 (6)	
H14A	0.3109	0.1434	0.0577	0.060*	
H14B	0.4985	0.1696	0.0824	0.060*	
C15	0.1637 (3)	0.05019 (11)	0.17242 (19)	0.0497 (6)	
H15A	0.1273	0.0125	0.2063	0.060*	
H15B	0.1937	0.0379	0.1038	0.060*	
C16	0.0165 (3)	0.09661 (13)	0.1536 (2)	0.0577 (6)	
H16A	0.0514	0.1342	0.1190	0.069*	
H16B	−0.0148	0.1089	0.2219	0.069*	
Cl1	1.09507 (11)	0.18434 (6)	0.87299 (8)	0.0971 (3)	
Cl2	0.43645 (12)	0.06673 (3)	0.87659 (6)	0.0720 (2)	
Cl3	−0.16257 (10)	0.06186 (4)	0.07135 (7)	0.0781 (3)	
Cl4	0.32971 (10)	0.22004 (3)	0.18815 (6)	0.0651 (2)	
N1	0.3156 (2)	0.07637 (9)	0.24030 (14)	0.0420 (4)	
N2	0.7258 (2)	0.19471 (9)	0.75320 (14)	0.0434 (4)	
O1	0.1805 (3)	0.06964 (11)	0.38336 (14)	0.0699 (6)	
O2	0.7529 (3)	0.26329 (8)	0.62021 (14)	0.0593 (5)	
S1	0.43739 (8)	0.16811 (3)	0.50083 (4)	0.04896 (18)	

### Atomic displacement parameters (Å^2^)

**Table:**

	*U*^11^	*U*^22^	*U*^33^	*U*^12^	*U*^13^	*U*^23^
C1	0.0423 (12)	0.0513 (12)	0.0326 (10)	0.0084 (9)	0.0052 (8)	0.0022 (9)
C2	0.0492 (13)	0.0534 (13)	0.0302 (10)	0.0059 (10)	0.0066 (9)	−0.0019 (9)
C3	0.0471 (13)	0.0543 (13)	0.0321 (10)	0.0109 (10)	0.0082 (9)	0.0025 (9)
C4	0.0547 (14)	0.0508 (12)	0.0307 (10)	0.0123 (10)	0.0089 (9)	0.0026 (9)
C5	0.0496 (15)	0.0781 (18)	0.0526 (14)	0.0201 (13)	0.0054 (11)	−0.0018 (13)
C6	0.0777 (19)	0.0630 (16)	0.0451 (13)	0.0261 (14)	0.0073 (12)	−0.0057 (11)
C7	0.0508 (14)	0.0507 (12)	0.0361 (11)	0.0023 (10)	0.0072 (10)	0.0025 (9)
C8	0.0369 (12)	0.0505 (12)	0.0390 (11)	0.0072 (9)	0.0058 (9)	0.0011 (9)
C9	0.0589 (15)	0.0538 (13)	0.0373 (11)	0.0106 (11)	0.0077 (10)	0.0037 (10)
C10	0.0720 (19)	0.0531 (14)	0.0737 (17)	0.0027 (13)	0.0295 (14)	0.0103 (13)
C11	0.0556 (16)	0.0686 (16)	0.0464 (13)	0.0011 (12)	0.0026 (11)	−0.0132 (12)
C12	0.068 (2)	0.096 (2)	0.079 (2)	−0.0103 (17)	−0.0062 (16)	−0.0104 (18)
C13	0.0453 (13)	0.0577 (13)	0.0357 (11)	0.0011 (10)	0.0093 (9)	−0.0033 (9)
C14	0.0542 (15)	0.0603 (14)	0.0376 (11)	−0.0078 (11)	0.0100 (10)	−0.0009 (10)
C15	0.0568 (15)	0.0478 (12)	0.0435 (12)	−0.0091 (11)	0.0049 (10)	−0.0084 (10)
C16	0.0510 (15)	0.0590 (15)	0.0592 (15)	−0.0086 (12)	−0.0024 (11)	−0.0083 (12)
Cl1	0.0548 (5)	0.1416 (9)	0.0898 (6)	0.0198 (5)	−0.0041 (4)	0.0276 (6)
Cl2	0.1030 (6)	0.0533 (4)	0.0654 (4)	−0.0134 (3)	0.0309 (4)	−0.0015 (3)
Cl3	0.0515 (4)	0.1027 (6)	0.0773 (5)	−0.0200 (4)	0.0019 (3)	−0.0212 (4)
Cl4	0.0750 (5)	0.0475 (3)	0.0758 (5)	−0.0069 (3)	0.0213 (4)	−0.0027 (3)
N1	0.0468 (11)	0.0451 (10)	0.0340 (9)	−0.0025 (8)	0.0056 (8)	−0.0029 (7)
N2	0.0432 (11)	0.0517 (10)	0.0346 (9)	0.0023 (8)	0.0037 (7)	−0.0030 (8)
O1	0.0591 (12)	0.1096 (17)	0.0431 (9)	−0.0159 (11)	0.0148 (8)	0.0033 (10)
O2	0.0674 (12)	0.0544 (10)	0.0566 (10)	−0.0052 (8)	0.0112 (9)	0.0066 (8)
S1	0.0447 (3)	0.0604 (4)	0.0402 (3)	0.0135 (3)	0.0020 (2)	−0.0095 (2)

### Geometric parameters (Å, °)

**Table:**

C1—C3	1.366 (3)	C10—H10A	0.9700
C1—C8	1.502 (3)	C10—H10B	0.9700
C1—S1	1.717 (2)	C11—N2	1.468 (3)
C2—C4	1.365 (3)	C11—C12	1.490 (4)
C2—C7	1.494 (3)	C11—H11A	0.9700
C2—S1	1.724 (2)	C11—H11B	0.9700
C3—C4	1.440 (3)	C12—Cl1	1.766 (4)
C3—C5	1.497 (3)	C12—H12A	0.9700
C4—C6	1.504 (3)	C12—H12B	0.9700
C5—H5A	0.9600	C13—N1	1.460 (3)
C5—H5B	0.9600	C13—C14	1.498 (3)
C5—H5C	0.9600	C13—H13A	0.9700
C6—H6A	0.9600	C13—H13B	0.9700
C6—H6B	0.9600	C14—Cl4	1.786 (3)
C6—H6C	0.9600	C14—H14A	0.9700
C7—O1	1.231 (3)	C14—H14B	0.9700
C7—N1	1.350 (3)	C15—N1	1.469 (3)
C8—O2	1.227 (3)	C15—C16	1.514 (4)
C8—N2	1.350 (3)	C15—H15A	0.9700
C9—N2	1.465 (3)	C15—H15B	0.9700
C9—C10	1.505 (4)	C16—Cl3	1.777 (3)
C9—H9A	0.9700	C16—H16A	0.9700
C9—H9B	0.9700	C16—H16B	0.9700
C10—Cl2	1.784 (3)		
			
C3—C1—C8	127.6 (2)	N2—C11—H11A	108.8
C3—C1—S1	112.81 (17)	C12—C11—H11A	108.8
C8—C1—S1	119.44 (16)	N2—C11—H11B	108.8
C4—C2—C7	131.1 (2)	C12—C11—H11B	108.8
C4—C2—S1	112.36 (17)	H11A—C11—H11B	107.7
C7—C2—S1	116.12 (17)	C11—C12—Cl1	112.3 (3)
C1—C3—C4	111.6 (2)	C11—C12—H12A	109.1
C1—C3—C5	124.5 (2)	Cl1—C12—H12A	109.1
C4—C3—C5	123.9 (2)	C11—C12—H12B	109.1
C2—C4—C3	112.1 (2)	Cl1—C12—H12B	109.1
C2—C4—C6	125.2 (2)	H12A—C12—H12B	107.9
C3—C4—C6	122.7 (2)	N1—C13—C14	114.0 (2)
C3—C5—H5A	109.5	N1—C13—H13A	108.7
C3—C5—H5B	109.5	C14—C13—H13A	108.7
H5A—C5—H5B	109.5	N1—C13—H13B	108.7
C3—C5—H5C	109.5	C14—C13—H13B	108.7
H5A—C5—H5C	109.5	H13A—C13—H13B	107.6
H5B—C5—H5C	109.5	C13—C14—Cl4	110.80 (15)
C4—C6—H6A	109.5	C13—C14—H14A	109.5
C4—C6—H6B	109.5	Cl4—C14—H14A	109.5
H6A—C6—H6B	109.5	C13—C14—H14B	109.5
C4—C6—H6C	109.5	Cl4—C14—H14B	109.5
H6A—C6—H6C	109.5	H14A—C14—H14B	108.1
H6B—C6—H6C	109.5	N1—C15—C16	112.64 (19)
O1—C7—N1	121.0 (2)	N1—C15—H15A	109.1
O1—C7—C2	119.5 (2)	C16—C15—H15A	109.1
N1—C7—C2	119.6 (2)	N1—C15—H15B	109.1
O2—C8—N2	122.0 (2)	C16—C15—H15B	109.1
O2—C8—C1	120.3 (2)	H15A—C15—H15B	107.8
N2—C8—C1	117.7 (2)	C15—C16—Cl3	110.22 (18)
N2—C9—C10	110.34 (19)	C15—C16—H16A	109.6
N2—C9—H9A	109.6	Cl3—C16—H16A	109.6
C10—C9—H9A	109.6	C15—C16—H16B	109.6
N2—C9—H9B	109.6	Cl3—C16—H16B	109.6
C10—C9—H9B	109.6	H16A—C16—H16B	108.1
H9A—C9—H9B	108.1	C7—N1—C13	124.28 (19)
C9—C10—Cl2	110.00 (19)	C7—N1—C15	117.58 (19)
C9—C10—H10A	109.7	C13—N1—C15	117.75 (17)
Cl2—C10—H10A	109.7	C8—N2—C9	123.80 (19)
C9—C10—H10B	109.7	C8—N2—C11	118.4 (2)
Cl2—C10—H10B	109.7	C9—N2—C11	117.63 (19)
H10A—C10—H10B	108.2	C1—S1—C2	91.11 (11)
N2—C11—C12	113.7 (2)		

### Hydrogen-bond geometry (Å, °)

**Table:**

*D*—H···*A*	*D*—H	H···*A*	*D*···*A*	*D*—H···*A*
C14—H14B···O2^i^	0.97	2.45	3.257 (3)	141
C14—H14A···Cl1^ii^	0.97	2.80	3.632 (3)	145
C6—H6B···O1^iii^	0.96	2.54	3.474 (3)	166
C5—H5B···O1^iv^	0.96	2.54	3.477 (3)	165

Symmetry codes: (i) *x*, −*y*+1/2, *z*−1/2; (ii) *x*−1, *y*, *z*−1; (iii) −*x*+1, −*y*, −*z*+1; (iv) *x*+1, *y*, *z*.
